# Appendico-Ileal Knotting (Appendiceal Tourniquet Syndrome): A Systematic Review and Clinicopathological Classification

**DOI:** 10.7759/cureus.102704

**Published:** 2026-01-31

**Authors:** Parmar Bhargav, Abhinav K Tiwari, Shailendra singh Nargesh, Komal Khuman, Raumil Parmar

**Affiliations:** 1 General Surgery, B. J. Medical College and Civil Hospital, Ahmedabad, IND; 2 General Surgery, Autonomous State Medical College, Firozabad, IND; 3 General Surgery, Government Medical College, Ratlam, IND; 4 Medicine, B. J. Medical College and Civil Hospital, Ahmedabad, IND; 5 Nephrology, Institute of Kidney Diseases and Research Centre (IKDRC), Ahmedabad, IND

**Keywords:** acute abdomen, appediceal torniquet syndrome, appendico-ileal knotting, closed-loop obstruction, computed tomography, exploratory laparotomy, small bowel obstruction

## Abstract

Appendico-ileal knotting (AIK), also known as appendiceal tourniquet syndrome, is an exceptionally rare and frequently overlooked cause of small bowel obstruction in which the appendix encircles the ileum, creating a closed-loop obstruction that rapidly progresses to ischemia and bowel gangrene. Despite advancements in cross-sectional imaging, AIK remains a formidable diagnostic enigma. Its clinical masquerade as common small bowel obstruction, coupled with the absence of pathognomonic radiographic markers, results in a persistently low preoperative diagnostic rate that necessitates a high index of clinical suspicion to avoid catastrophic intestinal ischemia. Delayed recognition often results in advanced bowel compromise and increased operative morbidity. This systematic review consolidates more than a century of published surgical experience to provide the most comprehensive clinicopathological synthesis of AIK to date. Thirty-two surgically confirmed cases were analyzed with respect to demography, clinical presentation, radiological features, operative management, and outcomes. The condition predominantly affected adults with a marked male preponderance, although recent reports demonstrate increasing recognition in younger patients and females without prior abdominal surgery. Clinically, patients most often presented with features of acute or subacute distal small bowel obstruction, while classical signs of appendicitis were inconsistently observed, contributing to diagnostic delay.

A consistent intraoperative finding across cases was the high incidence of bowel ischemia and gangrene at exploration, underscoring the aggressive nature of this pathology. While conventional radiography remained nonspecific, cross-sectional imaging demonstrated improved diagnostic yield when characteristic closed-loop obstruction patterns were identified. Surgical management was definitive, ranging from simple appendectomy with untwisting in selected viable cases to en bloc bowel resection with anastomosis or stoma formation depending upon the status of the affected segment of bowel. Despite advances in imaging and minimally invasive techniques, AIK continues to be associated with substantial morbidity and non-negligible mortality. Based on cumulative clinical, radiological, and operative insight, this review proposes a practical clinicopathological framework distinguishing inflammatory and mechanical subtypes of AIK. This distinction provides valuable insight into disease pathogenesis, explains variations in presentation, and has direct implications for surgical decision-making. The primary merit and novelty of this systematic review lie in its transition of AIK from an anecdotal surgical anomaly into a structured, classifiable clinical entity, rather than viewing AIK as a mere intraoperative surprise. Heightened awareness, early imaging, and prompt surgical intervention are essential to improving outcomes in this rare but life-threatening condition.

## Introduction and background

Appendico-ileal knotting (AIK) is an exceptionally rare surgical entity in which the appendix encircles the terminal ileum or its mesentery, resulting in a closed-loop small-bowel obstruction (SBO) with a high risk of strangulation and ischemia [1]. Because of its infrequent occurrence and nonspecific presentation, the condition remains poorly recognized and is often diagnosed only at the time of exploration.

The earliest description of this phenomenon was provided by Hotchkiss in 1901, who reported a case of intestinal obstruction secondary to appendicitis identified during exploratory laparotomy [2]. For many decades thereafter, AIK was considered an unusual complication of untreated or advanced appendicitis. More recent case reports, however, have expanded this understanding by describing cases in which a non-inflamed but elongated and mobile appendix acts as a constricting loop around the ileum, thereby establishing a mechanical mechanism distinct from inflammatory pathology [3-9].

SBO itself is a frequent cause of emergency surgical admission worldwide, most commonly resulting from postoperative adhesions, hernias, malignancy, or inflammatory strictures [10]. Internal strangulation in AIK represents a diagnostic challenge, particularly in patients without prior abdominal surgery [11].

The pathogenesis of AIK is thought to involve either inflammatory adhesions related to acute appendicitis or anatomical variations such as excessive appendicular length and mobility [12,13]. Mechanical forms of AIK often lack classical signs of inflammation, which contributes to delayed recognition and increases the likelihood of bowel ischemia and gangrene at presentation.

In view of its rarity, fragmented reporting, and diagnostic complexity, a comprehensive synthesis of available evidence is required. This systematic review aims to consolidate historical and contemporary literature on AIK, propose a practical clinicopathological classification, and highlight key diagnostic and surgical considerations relevant to modern clinical practice. Unlike prior reports that describe AIK as isolated surgical curiosities, this systematic review represents the first comprehensive synthesis of published cases spanning more than a century and proposes a unified clinicopathological classification integrating clinical, radiological, and operative findings.

Materials and methods

**Figure 1:**
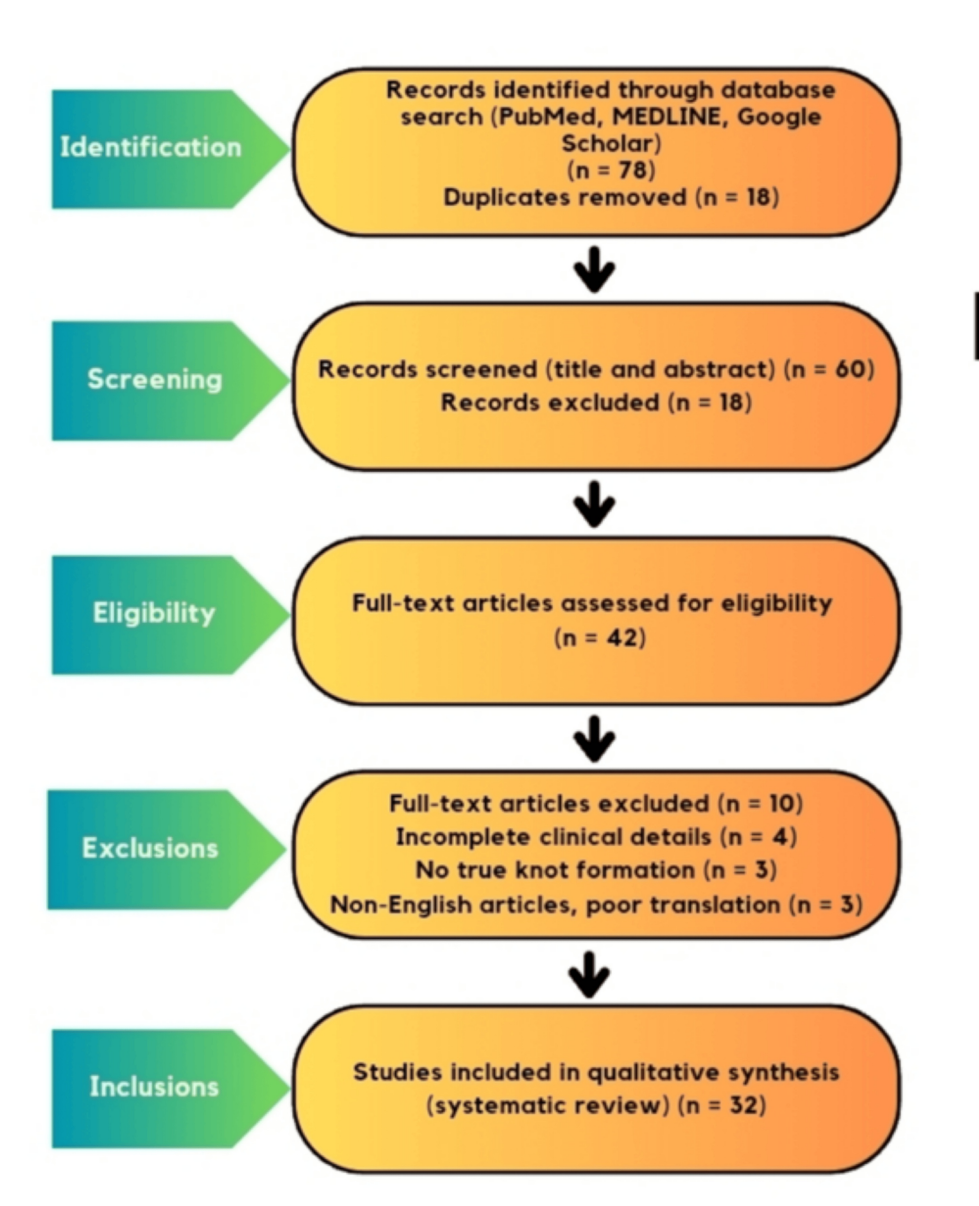
PRISMA 2020 flow diagram. PRISMA 2020 flow diagram illustrating the study selection process for the systematic review of appendico-ileal knotting [14]. PRISMA, Preferred Reporting Items for Systematic Reviews and Meta-Analyses

This systematic review was conducted and reported in accordance with the Preferred Reporting Items for Systematic Reviews and Meta-Analyses (PRISMA) 2020 guidelines [14]. The study selection process, including identification, screening, eligibility assessment, and inclusion of studies, is summarized using a PRISMA 2020 flow diagram (Figure 1).

Search strategy

A comprehensive literature search was performed using PubMed, MEDLINE, and Google Scholar for articles published between January 1901 and December 2025. Search terms included “appendico-ileal knotting,” “appendicular knot,” “appendiceal tie syndrome,” and “appendix causing small bowel obstruction.”

Studies were included if they reported a surgically confirmed intraoperative diagnosis of AIK with full-text availability and provided adequate documentation of clinical presentation, operative management, and postoperative outcomes.

Studies were excluded if operative details were incomplete, if articles were published in non-English languages without reliable translation, or if bowel obstruction was attributed to appendiceal pathology without true knot formation.

A total of 32 cases met the inclusion criteria. Extracted variables included patient condition demographics, history and clinical findings, appendix status, ileal viability, imaging findings, operative procedure, and outcomes. Data analysis was descriptive due to the rarity of the AIK and the nature of available evidence.

Study selection and data extraction were independently performed by two authors, and disagreement resolved by consensus.

## Review

AIK is an exceptionally rare cause of SBO. Since its first description by Hotchkiss in 1901, fewer than 100 cases have been documented worldwide, underscoring the rarity of this entity [2]. Contemporary reports consistently demonstrate a male predominance, with an estimated male-to-female ratio of approximately 3:1, accounting for nearly 75% of reported cases [2,3]. Despite this historical trend, recent literature has highlighted an increasing number of cases in young females without prior abdominal surgery, suggesting that the role of anatomical factors is underestimated [4,5]. Clinically, AIK most often presents with features of acute or subacute distal SBO, including colicky abdominal pain, vomiting, abdominal distension, and obstipation [6]. Unlike classical inflammatory abdominal conditions, systemic signs such as fever and leukocytosis are inconsistently present, particularly when the appendix is not inflamed. This atypical presentation frequently contributes to delayed diagnosis and late surgical intervention [7]. The natural course of AIK is often aggressive due to the formation of a closed-loop obstruction, predisposing the involved bowel segment to rapid strangulation and ischemia. Several reports describe progression to bowel compromise within 24-72 hours of symptom onset [8]. As a result, 65% of reviewed cases demonstrated gangrenous ileum at the time of Exploration, necessitating bowel resection and anastomosis or stoma formation rather than simple untwisting [9]. Operative findings typically include a loop of terminal ileum encircled by the appendix or its mesentery, with variable degrees of vascular compromise. The appendix may appear inflamed, gangrenous, or completely normal, depending on the underlying mechanism [10].

The key clinical characteristics, radiological findings, operative management, and outcomes of all reported cases included in this review are summarized in Table 1

**Table 1:** Comprehensive analysis of AIK literature and clinical outcomes (1901-2025). CT, computed tomography; AIK, appendico-ileal knotting

Study year	Age/Sex	Appendix status	Ileum status	Key radiological sign	Surgical procedure	Outcome
Kabuye et al. (2024) [1]	28 years/female	Inflamed	Gangrenous	Dilated bowel loops [1,10]	Right hemicolectomy	Recovered
Hotchkiss (1901) [2]	Adult/male	Inflamed	Gangrenous	Based on clinical suspicion [2]	Open resection with anastomosis	Recovered
Mandal and Zenebe (2025) [3]	64 years/male	Healthy	Gangrenous	Whirl sign on CT [6]	Resection and anastomosis	Recovered
Ohenewaa et al. (2025) [4]	28 years/female	Healthy	Gangrenous	Multiple air fluid level [10]	Ileocecal resection	Recovered
Soressa and Datiko (2025) [5]	15 years/female	Inflamed	Viable	Medial cecum sign [6]	Appendectomy and untwisting	Recovered

New pathophysiological framework

Based on cumulative evidence from published cases, AIK can be conceptually divided into two distinct pathophysiological subtypes: inflammatory and mechanical. This classification shares important diagnostic and therapeutic implications. Inflammatory AIK occurs secondary to acute appendicitis, where inflammation, phlegmon formation, or adhesions create a rigid constricting ring around the ileum. This mechanism represents the classical form originally described in early literature and is often associated with systemic inflammatory signs [11,12]. In contrast, mechanical AIK arises in the absence of appendiceal inflammation and is primarily driven by anatomical variation, most notably a long and mobile appendix acting as a dynamic tourniquet around the small bowel or its mesentery. This subtype frequently presents in patients with a virgin abdomen and minimal inflammatory response, making preoperative diagnosis particularly challenging [13,14,15]. 

Table2 provides the first unified clinicopathological classification of AIK, translating scattered case-based evidence into a practical diagnostic and management framework.

**Table 2:** Proposed clinicopathological classification and diagnostic framework for AIK. AIK, appendico-ileal knotting; SBO, small-bowel obstruction; RIF, right iliac fossa

Features	Inflammatory AIK	Mechanical AIK
Primary etiology	Acute appendicitis/adhesions [8,12]	Anatomical variation (long appendix) [3,5,13]
Clinical hallmark	Fever, leukocytosis, and RIF pain [11,12,13]	SBO in the virgin abdomen [9,16]
Pathophysiology	Phlegmon creating a rigid ring or adhesions [8,12]	Long appendix acting as a tourniquet [3,5,15]
Key CT findings	Inflamed appendix with wall thickening [8,12]	Whirl sign, medial cecum displacement [6]
Histology	Suppurative or gangrenous [1,3,5]	Histologically normal tissue [16]
Definitive treatment	En bloc resection [9]	Untwisting if possible [16]

Plain abdominal radiographs are generally nonspecific and typically demonstrate features consistent with SBO. Computed tomography (CT) has emerged as the diagnostic imaging modality of choice for preoperative assessment. Among CT findings, the ‘whirl sign’ representing twisted bowel loops and mesenteric vessels at the point of torsion is the most reliable indicator of a closed-loop obstruction and impending strangulation [13,15,16]. An additional, more subtle radiological feature is medial displacement of the cecum, caused by traction from the appendiceal loop; the cecum itself is not medially rotated. Recognition of this finding may aid in distinguishing AIK from other causes of right-sided SBO [17,18]. Despite these advances, definitive diagnosis is still commonly made intraoperatively [19].

Discussion

AIK remains underrecognized due to its nonspecific presentation. The increasing documentation of mechanical AIK challenges the traditional assumption that SBO in a virgin abdomen is benign or adhesive in nature [20,21].

Surgical management is definitive. When bowel viability is preserved, appendectomy with gentle untwisting may be sufficient [22]. However, in cases of suspected gangrene, en bloc resection without detorsion is recommended to prevent systemic toxin release and reperfusion injury [23,24]. Diagnostic laparoscopy has emerged as a valuable tool for early diagnosis and management in selected cases.

Limitations

This review is limited by its reliance on case reports and small series, which restricts statistical analysis and generalizability. Inconsistent imaging and operative descriptions in historical literature further limit standardized comparison.

Uniqueness and strengths

This study represents the most comprehensive review of AIK spanning over 120 years. It is the first to formally distinguish mechanical AIK as a separate clinicopathological entity and propose a unified classification system integrating clinical, radiological, and operative findings. By consolidating historical and contemporary data, this review transforms AIK from a surgical curiosity into a recognizable and clinically relevant syndrome.

## Conclusions

AIK is a rare but life-threatening cause of small bowel obstruction that remains difficult to diagnose preoperatively due to its nonspecific clinical presentation. AIK occurs through two distinct mechanisms: an inflammatory subtype and a mechanical subtype caused by anatomical variations such as a long, mobile appendix. Recognition of these subtypes is clinically important, as mechanical cases frequently present without inflammatory signs and often occur in patients with a virgin abdomen, contributing to delayed diagnosis and increased risk of bowel ischemia. CT is the diagnosis of choice, with findings such as the *whirl sign* offering important clues; however, definitive diagnosis is usually made intraoperatively. Surgical management is mandatory and should be guided by bowel viability, ranging from appendectomy with untwisting to en bloc resection in cases of gangrene. The principal strength of this review lies in its proposed clinicopathological classification, which integrates clinical presentation, radiological features, and operative strategy into a practical framework. By transforming isolated case reports into a structured and applicable model, this study enhances awareness of AIK and supports earlier recognition and timely surgical intervention, with the potential to reduce morbidity and mortality.
